# Lysyl Oxidase Mediates Proliferation and Differentiation in the Esophageal Epithelium

**DOI:** 10.3390/biom14121560

**Published:** 2024-12-07

**Authors:** Kanak V. Kennedy, Joshua X. Wang, Emily McMillan, Yusen Zhou, Ryugo Teranishi, Ann Semeao, Leena Mirchandani, Chizoba N. Umeweni, Diya Dhakal, Alyssa Baccarella, Satoshi Ishikawa, Masaru Sasaki, Takefumi Itami, Adele C. Harman, Leonel Joannas, Tatiana A. Karakasheva, Hiroshi Nakagawa, Amanda B. Muir

**Affiliations:** 1 Division of Gastroenterology, Hepatology, and Nutrition, Children’s Hospital of Philadelphia, Philadelphia, PA 19104, USAmcmillanea@chop.edu (E.M.); zhouy10@chop.edu (Y.Z.); teranishir@chop.edu (R.T.); aes432@cornell.edu (A.S.); lmirchandani-25@peddie.org (L.M.); dhakald@chop.edu (D.D.); baccarella@chop.edu (A.B.); ishikawas1@chop.edu (S.I.); masarusan1220@gmail.com (M.S.); itamit@chop.edu (T.I.); karakashet@chop.edu (T.A.K.); 2 Division of Pediatric Gastroenterology, Hepatology, and Nutrition, Department of Pediatrics, Stanford University School of Medicine, Palo Alto, CA 94304, USA; 3 Department of Pediatrics, Children’s Hospital of Philadelphia, Philadelphia, PA 19104, USA; 4 Transgenic Core, CHOP Research Institute, Children’s Hospital of Philadelphia, Philadelphia, PA 19104, USA; harmana@chop.edu; 5 Department of Pathology and Laboratory Medicine, Perelman School of Medicine, University of Pennsylvania, Philadelphia, PA 19104, USA; ljoannas@pennmedicine.upenn.edu; 6 Division of Digestive and Liver Diseases, Department of Medicine and Herbert Irving Comprehensive Cancer Center, Columbia University Irving Medical Center, New York, NY 10032, USA; hn2360@cumc.columbia.edu

**Keywords:** lysyl oxidase, esophagus, epithelial homeostasis, proliferation, differentiation

## Abstract

In homeostatic conditions, the basal progenitor cells of the esophagus differentiate into a stratified squamous epithelium. However, in the setting of acid exposure or inflammation, there is a marked failure of basal cell differentiation, leading to basal cell hyperplasia. We have previously shown that lysyl oxidase (LOX), a collagen crosslinking enzyme, is upregulated in the setting of allergic inflammation of the esophagus; however, its role beyond collagen crosslinking is unknown. Herein, we propose a non-canonical epithelial-specific role of LOX in the maintenance of epithelial homeostasis using 3D organoid and murine models. We performed quantitative reverse transcriptase PCR, Western blot, histologic analysis, and RNA sequencing on immortalized non-transformed human esophageal epithelial cells (EPC2-hTERT) with short-hairpin RNA (shRNA) targeting *LOX* mRNA in both monolayer and 3D organoid culture. A novel murine model with a tamoxifen-induced Lox knockout specific to the stratified epithelium (*K5CreER*; *Lox^fl/fl^)* was utilized to further define the role of epithelial LOX in vivo. We found that LOX knockdown decreased the proliferative capacity of the esophageal epithelial cells in monolayer culture, and dramatically reduced the organoid formation rate (OFR) in the shLOX organoids. LOX knockdown was associated with decreased expression of the differentiation markers filaggrin, loricrin, and involucrin, with RNA sequencing analysis revealing 1224 differentially expressed genes demonstrating downregulation of pathways involved in cell differentiation and epithelial development. Mice with Lox knockout in their stratified epithelium demonstrated increased basaloid content of their esophageal epithelium and decreased Ki-67 staining compared to the vehicle-treated mice, suggesting reduced differentiation and proliferation in the Lox-deficient epithelium in vivo. Our results demonstrate, both in vivo and in vitro, that LOX may regulate epithelial homeostasis in the esophagus through the modulation of epithelial proliferation and differentiation. Understanding the mechanisms of perturbation in epithelial proliferation and differentiation in an inflamed esophagus could lead to the development of novel treatments that could promote epithelial healing and restore homeostasis.

## 1. Introduction

The esophageal mucosa consists of a complex epithelium that aims to maintain homeostasis in the setting of repeated exposure to both antigenic and biophysical stimuli [1]. This multi-layered squamous epithelium is maintained through a finely regulated proliferation–differentiation gradient, in which proliferative basal cells migrate towards the lumen and execute a terminal differentiation program [2,3,4]. These differentiating cells give rise to a supra-basal cell layer and a terminally differentiated superficial cell layer [5,6]. This gradient is essential for epithelial barrier function and is perturbed in several esophageal pathologies [7], including gastroesophageal reflux disease (GERD) [8], eosinophilic esophagitis (EoE) [9,10], and Barrett’s esophagus [11,12].

Lysyl oxidase (LOX) is an extracellular matrix remodeling enzyme that covalently crosslinks lysine residues on collagen molecules, allowing for the stabilization of its component chains and subsequent collagen fibril development [13]. LOX is essential for the structural integrity and function of collagen, providing tensile strength and elastic properties for various extracellular matrices, including those in lung tissue, vascular walls, and skin [14,15]. Global murine Lox deletion has been shown to cause structural alterations in arterial walls, leading to aneurysm development and perinatal death in mouse models [16,17]. While its role as a collagen crosslinker that promotes fibrosis has been appreciated, there is a growing body of literature supporting the non-collagen crosslinking role of LOX in chemotaxis, gene regulation, cell proliferation and differentiation, and oncogenesis [18,19,20,21,22,23]. In keratinocytes, LOX silencing by RNA interference is associated with decreased expression of terminal markers of differentiation involucrin (IVL) and keratin 10 (KRT10) in vitro [24]. We have previously shown that in vitro LOX overexpression in esophageal epithelial cells restores epithelial differentiation and barrier integrity, including in the setting of IL-13-induced disruption [25]. While this suggests a role for LOX in squamous differentiation and epithelial homeostasis, experimental studies evaluating LOX in both the esophageal epithelium and skin have been limited to in vitro models. A Lox knockout in vivo remains challenging given the lethality of global deletion [16].

To further understand the functional role of LOX in the esophageal epithelium, we conducted parallel studies employing in vitro (human) and in vivo (murine) models of LOX silencing. Herein, we describe a novel model supporting that LOX has an essential role in epithelial proliferation and differentiation in the esophageal epithelium.

## 2. Materials and Methods

### 2.1. Cell Line and Monolayer

Immortalized non-transformed esophageal epithelial cells (EPC2-hTERT) [26] were cultured in a keratinocyte serum-free medium containing 0.09 mM Ca^2+^ (KBM-Gold Keratinocyte Growth Medium, Catalog #192060, Lonza, Basel, Switzerland). The cells were incubated at 37 °C in a humidified 95% O_2_ and 5% CO_2_ atmosphere.

### 2.2. Lentivirus-Mediated Gene Transfer

Mission lentiviral transduction particles were utilized to construct a tetracycline-inducible short-hairpin RNA-targeting LOX (Sigma-Aldrich, St. Louis, MA, USA). The Tet-pLKO-puro lentiviral vector expressed a tetracycline-inducible short-hairpin RNA (shRNA) sequence targeting LOX mRNA (shLOX, TRCN00000293943), or a scramble shRNA (shSCR, SHC002) as a control sequence, activating the RNA interference pathway without targeting any other known mRNA. The transfection was performed on subconfluent EPC2-hTERT cells by spinfection at 800 g for 1 h at room temperature in keratinocyte media supplemented with 8 μg/mL Polybrene, and selected for with 10 μg/mL blasticidin for three continuous cell passages.

### 2.3. WST-1 Assay for Cell Viability and Proliferation

The shSCR and shLOX cells were seeded in a 96-well plate at a concentration of 5000 cells per well and cultured in keratinocyte media containing doxycycline to induce shRNA targeted to scramble mRNA and *LOX* mRNA, respectively, for 24 h. Cellular viability was assessed utilizing a water-soluble tetrazolium (WST-1, tetrazolium salt 4-[3-(4-Iodophenyl)-2-(4-nitro-phenyl)-2H-5-tetrazolio]-1,3-benzene sulfonate) assay (Millipore Sigma, Burlington, MA, USA) according to the manufacturer’s instructions. Briefly, the cells were incubated with 10 μL/well of WST-1 reagent in 100 μL keratinocyte media for 30 min at 37 °C and 5% CO_2_. The absorbance of the samples was measured at a wavelength of 480 mm against a background control using a scanning multi-well spectrophotometer.

### 2.4. Three-Dimensional Esophageal Organoid Culture

**Figure 1:**
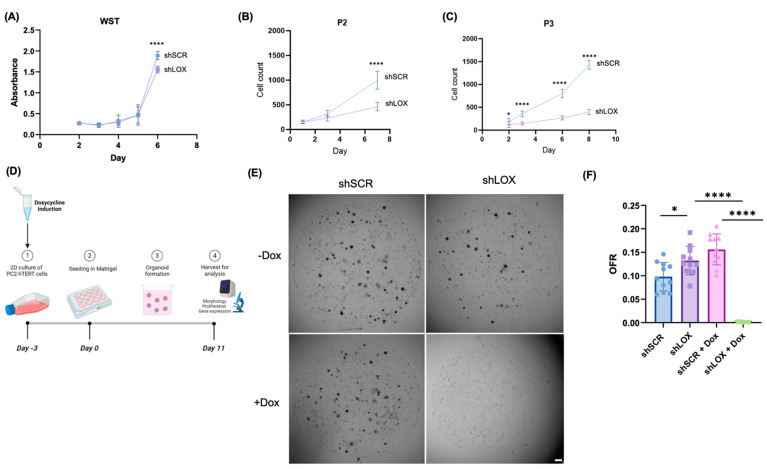
LOX knockdown decreases proliferative capacity of esophageal epithelium. (**A**) Metabolic activity of subconfluent shSCR and shLOX EPC2-hTERT cells grown in monolayer culture with continuous doxycycline exposure, based on water-soluble tetrazolium salt (WST-1) assay. (**B**,**C**) Cell counts following second passage and third passage, respectively, for shSCR and shLOX cells grown in monolayer culture with continuous doxycycline exposure. (**D**) Schematic illustrating protocol for doxycycline-treated shSCR and shLOX cells in monolayer culture beginning on day -3, with subsequent organoid seeding on day 0. Organoids were grown in the presence or absence of continuous doxycycline and were harvested on day 11 for analysis. (**E**) Representative phase contrast images of shSCR and shLOX organoids on day 11 with and without continuous doxycycline. Scale bar, 1 mm. (**F**) Organoid formation rate (OFR) for shSCR and shLOX organoids grown with and without continuous doxycycline. OFR was assessed on day 11 and was calculated as number of organoids (≥50 μm) divided by number of total seeded cells. Data illustrated are representative of three independent experiments and expressed as means ± SDs. **** *p* < 0.0001; * *p* < 0.05.

**Figure 2:**
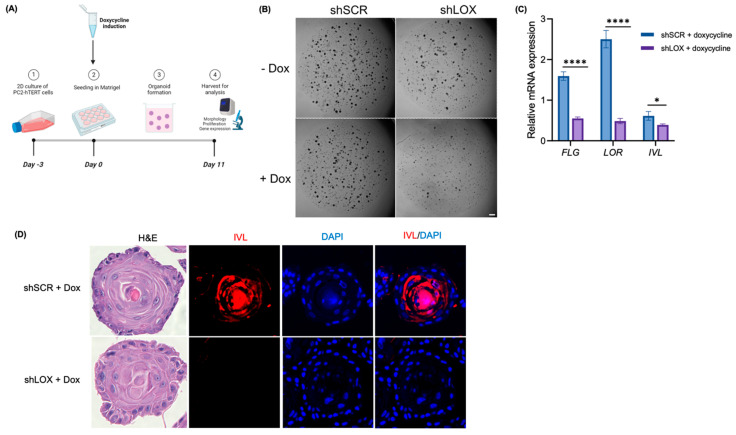
LOX knockdown alters esophageal epithelial differentiation in vitro. (**A**) Schematic illustrating protocol for 3D organoids grown from shSCR and shLOX cells in monolayer culture. Doxycycline exposure was initiated on day 0 at the time of organoid seeding. Organoids were grown in the presence or absence of continuous doxycycline and were harvested on day 11 for analysis. (**B**) Representative phase contrast images of shSCR and shLOX organoids with and without continuous doxycycline on day 11. Scale bar, 1 mm. (**C**) qRT-PCR for filaggrin (*FLG*), loricrin (*LOR*), and involucrin (*IVL*) mRNA expression in shSCR and shLOX organoids treated with continuous doxycycline. (**D**) Representative images of H&E staining and immunofluorescence staining for IVL (red) in shSCR and shLOX organoids treated with continuous doxycycline. Data illustrated are representative of three independent experiments and expressed as means ± SDs. **** *p* < 0.0001; * *p* < 0.05.

We evaluated the effects of LOX knockdown prior to and after the establishment of the organoid culture. The shSCR and shLOX organoids were cultured as described previously [27]. Briefly, the cells were dissociated into a single-cell suspension. The cell number and viability were measured using an Automated Cell Counter (Invitrogen Countess II, Bothell, WA, USA), by mixing 5 μL of cell suspension with 5 μL of 0.4% trypan blue solution (T10282; Invitrogen; Eugene, OR, USA). The cells were then suspended in Matrigel basement membrane extract (354234; Corning Inc., Corning, NY, USA), seeded as 2000 live cells per 50 μL Matrigel dome. The cells were cultured in keratinocyte serum-free medium (KBM-Gold Keratinocyte Growth Medium, Catalog #192060, Lonza, Basel, Switzerland) with the addition of 0.6 mM Ca^2+^. To evaluate organoid formation in the setting of LOX knockdown, we treated the shSCR and shLOX EPC2-hTERT cells with doxycycline (10 ng/mL) for three days prior to seeding the organoid culture (see schematic Figure 1D). In addition, we evaluated the effect of LOX knockdown on organoid growth by adding doxycycline (10 ng/mL) on day 0 of the organoid culture (see schematic Figure 2A). The day 11 organoids were used for all further analyses. The organoid formation rate (OFR) was defined as the number of organoids ≥50 μm divided by the total seeded cells in each well [28].

### 2.5. Quantitative Reverse Transcription-Polymerase Chain Reaction

RNA extraction and reverse transcription were performed as previously described [29,30]. The real-time qRT-PCR was performed with TaqMan Gene Expression Assays (Thermo Fisher Scientific Inc., Waltham, MA, USA) for the human genes LOX (Hs00942480_m1), IVL (Hs00846307_s1), filaggrin (FLG, Hs00856927_g1), loricrin (LOR, Hs01894962_s1), and glyceraldehyde-3-phosphate dehydrogenase (GAPDH, Hs02786624_g1), as well as for the mouse genes Lox (Mm00495386_m1) and Gapdh (Mm99999915_g1) using the StepOnePlus Real-Time PCR System (Catalog Number #4376600) (Thermo Fisher Scientific Inc. Waltham, MA, USA). The relative mRNA levels for each gene were normalized to the GAPDH mRNA levels as a housekeeping control.

### 2.6. Western Blot

Whole-cell lysates from the cells in monolayer culture were prepared as previously described [25,28]. Equivalent amounts (20–40 μg) of protein for each sample were loaded onto a NuPAGE 4 to 12% Bis-Tris gel. Following electrophoresis, transfer to a polyvinylidene difluoride membrane, and blocking with 5% bovine serum albumin or non-fat milk, the membranes were incubated with primary antibodies at 4 °C overnight. The primary antibodies used were anti-LOX (1:100; R36430; NSJ Bioreagents, San Diego, CA, USA) and anti-β-actin (1:5000; A5316; Millipore Sigma, Burlington, MA, USA). The immunoblots were detected with an appropriate horseradish peroxidase (HRP)-conjugated secondary antibody (1:2000; NA934 or NA 931; Amersham BioSciences, Buckinghamshire, UK) using Clarity Max ECL Substrate (1704272; Bio-Rad Laboratories, Hercules, CA, USA). β-actin served as the loading control.

### 2.7. Immunohistochemistry and Immunofluorescence

The organoids were fixed and embedded as previously described [27], and subjected to hematoxylin and eosin (H&E) staining, immunohistochemistry (IHC), and immunofluorescence (IF).

The IHC of the murine esophageal specimens was performed using a Leica Bond RXm (Leica Biosystems, Nusslock, Germany) with an anti-Ki-67 antibody (1:200; ab15580; Abcam, Cambridge, UK).

For the IF, the sections were stained with primary anti-IVL monoclonal antibody (1:100, I9018; Millipore Sigma) at 4 °C overnight and then with Cy5 AffiniPure Donkey Anti-Mouse IgG (H+L) secondary antibody (1:500; 715-175-150; Jackson ImmunoResearch, West Grove, PA, USA) at room temperature for 1 h. The nuclei were stained with 4′,6-diamidino-2-phenylindole (DAPI; 17985-50; Electron Microscopy Sciences, Hatfield, PA, USA).

Imaging was performed with a BZ-X710 Fluorescence Microscope (Keyence, Osaka, Japan). All the sections were evaluated at 5 different locations in a high-power field per condition, and the representatives are shown.

The esophageal basaloid cell content was defined as the percentage of the total height of the basaloid cell layers per esophageal diameter, spanning both the basaloid cell layers and the differentiated cell layers, including the keratinized inner cell mass in the H&E-stained esophageal samples, with 3 areas measured per sample [28].

### 2.8. RNA Sequencing and Gene Expression Analysis

The shSCR and shLOX organoids were grown for 11 days in the presence or absence of doxycycline, and then harvested for RNA sequencing. The sequencing libraries were constructed from the total RNA (1 μg) using a TruSeq Stranded mRNA Library Prep (Illumina Inc., San Diego, CA, USA). The RNA sequencing was performed on the Illumina HiSeq2000 platform. We used the transcriptome pseudoalignment program kallisto (version 0.46.2) [31] with the default settings and the human reference genome hg38 for alignment. The mapped reads were input into R (v4.1.3). The read counts were normalized, and the differentially expressed genes (DEGs) were identified using the DESeq2 package (version 1.34.0) [32]. The DEGs were determined as *p* < 0.05 and log2 (fold change) ≥0.50 or ≤−0.50. Using the ‘stat’ output field of the DEGs from DESeq2, a Gene Ontology enrichment analysis was performed by topGO package [33]. The top enriched or depleted terms in the shLOX organoids were selected. The expression patterns for the genes included in the Gene Set Enrichment Analysis (GSEA) [34,35] epithelial cell differentiation gene set were compared between the shSCR and shLOX organoids.

### 2.9. Murine Model

We generated *Lox^fl/fl^* mice expressing tamoxifen-inducible Cre recombinase (CreER) under the control of the estrogen-responsive keratin 5 (*KRT5*) promoter using small knock-in insertions with CRISPR-Cas9 technology (Strain #029155; Jackson Laboratory, Bar Harbor, ME, USA). We employed murine zygote electroporation as a means to deliver the SpCas9 WT protein inserted into the target 5′ and 3′ sequences within the *Lox* locus [36]. The in vitro fertilization of sperm from a wild-type C57BL/6J male and oocytes from C57Bl/6J females was accomplished using the method of the Center for Animal Resources and Development (CARD), as previously described [37]. The founder mice underwent Sanger sequencing of the targeted allele PCR product to confirm the 5′ and 3′ *loxP* sites. These mice were then bred with *K5CreER* mice for a final genotype of *K5CreER+/−; Lox^fl/fl^*. The *K5CreER+/+; Lox+/+* mice were used as the control.

To induce Cre-mediated recombination, mice aged 8 weeks were injected intraperitoneally with 100 mg/kg body weight/dose of tamoxifen suspended in corn oil for 5 days, then injected with 50 mg/kg body weight/dose of tamoxifen for an additional 2 days. The mice who demonstrated more than 1 g of weight loss over the prior 24 h did not receive a scheduled tamoxifen dose. All the mice were euthanized on day 10. Each mouse’s esophagus was harvested for RNA and histology, and each mouse’s ventral skin was harvested for histology only. All the experiments were approved by the Children’s Hospital of Philadelphia Institutional Animal Care and Use Committee (IACUC) (Approval number IAC 22-001454, approved 17 October 2022).

### 2.10. Statistical Analysis

The data are presented as means ± standard deviations (SDs). The continuous variables were analyzed by a two-tailed Student’s *t*-test for two independent groups or an analysis of variance for comparing more than two groups. All the statistical analyses were conducted with GraphPad Prism Version 10.4.0 (GraphPad Software, San Diego, CA, USA). A *p* value of less than 0.05 was considered statistically significant.

## 3. Results

### 3.1. LOX Knockdown Decreases Proliferative Capacity and Organoid Formation Capacity of Esophageal Epithelium

We utilized RNA interference via doxycycline-inducible short-hairpin RNA to investigate the role of epithelial-derived LOX in vitro in the immortalized non-transformed esophageal epithelial progenitor cell line EPC2-hTERT (referred to as shSCR or shLOX below). LOX knockdown following three days of continuous doxycycline exposure was verified by a qRT-PCR (Supplementary Figure S1a) and Western blot (Supplementary Figure S1b).

We then utilized a WST-1 assay to detect differences in metabolic activity between the shSCR and shLOX cells. The shLOX cells with continuous doxycycline induction demonstrated decreased metabolic activity at day 6 when compared to the shSCR cells exposed to continuous doxycycline (Figure 1A).

As absorbance measured by WST-1 assay can serve as a proxy for cell proliferation, we next quantified cell proliferation in the setting of doxycycline-induced LOX knockdown to determine whether this is impacted by decreased LOX expression. The shLOX demonstrated significantly reduced cell counts by day 7 during the second passage (Figure 1B) and by day 2 during the third passage, with the continuous induction of the shRNA with doxycycline (Figure 1C).

We then investigated the impact of LOX knockdown in three-dimensional (3D) organoid culture [27]. The shSCR and shLOX cells were pre-treated in a 2D cell culture (see schematic Figure 1D) with doxycycline-containing keratinocyte media for three days prior to organoid formation to induce knockdown at the time of organoid seeding. Early LOX knockdown resulted in a lack of organoid formation (Figure 1E,F).

Taken together, these results suggest that LOX knockdown decreases the proliferative capacity of and organoid formation by esophageal epithelial cells in vitro.

### 3.2. LOX Knockdown Decreases Epithelial Differentiation In Vitro

In order to evaluate the effects of LOX knockdown on the esophageal epithelial architecture, we seeded organoids and added doxycycline to the organoid culture media at seeding (day 0) to allow for organoid formation prior to LOX knockdown (see schematic Figure 2A). We found decreased organoid formation in the setting of LOX knockdown, even in the organoids treated with doxycycline on the day of seeding (Figure 2B). In addition, the organoids that did form demonstrated decreased expression of markers of differentiation, including *FLG, LOR*, and *IVL* (Figure 2C). The H&E staining revealed an altered morphology in the LOX knockdown organoids, with absent IVL staining via immunofluorescence (Figure 2D). These findings indicate that LOX knockdown perturbs the differentiation of the esophageal epithelium.

### 3.3. RNA Sequencing of Epithelial Cells with LOX Knockdown Reveals Alterations in Epithelial Differentiation

**Figure 3:**
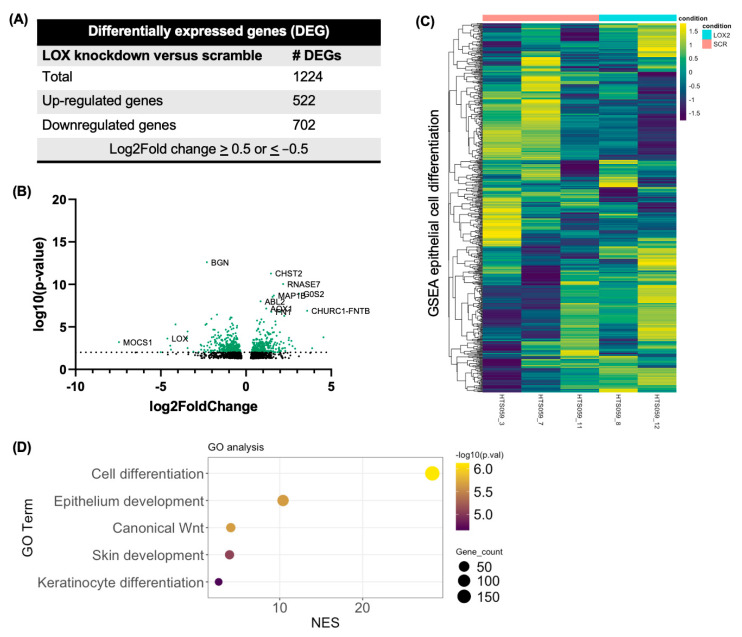
Transcriptomic analysis reveals the potential role of LOX in epithelial differentiation. (**A**) Total number of differentially expressed genes (DEGs) in shLOX organoids versus shSCR organoids treated with continuous doxycycline, based on log_2_ fold change of 0.5. (**B**) Volcano plot of all DEGs in shLOX organoids versus shSCR organoids treated with continuous doxycycline with log10 (*p*-value) > 2.0. (**C**) Heat map of RNA sequencing expression data for 757 genes included in gene set enrichment analyses (GSEAs) for epithelial cell differentiation. (**D**) Select Gene Ontology (GO) terms enriched in the downregulated DEGs. NES, normalized enrichment score.

To evaluate the global effects of LOX knockdown in esophageal epithelial culture, we grew shSCR and shLOX organoids as above, isolated the RNA, and subjected it to sequencing. A DESeq2 analysis identified 1224 unique differentially expressed genes (*p* < 0.05 and log_2_ fold change ≥ 0.50 or ≤ −0.50) in the doxycycline-induced shLOX organoids when compared to the doxycycline-induced shSCR organoids (Figure 3A,B). Figure 3C illustrates the RNA expression data from shSCR and shLOX organoids for the 757 genes included in the gene set enrichment analyses (GSEAs) for epithelial cell differentiation. A Gene Ontology (GO) analysis was performed on downregulated DEGs in the setting of LOX knockdown (log_2_ fold change ≤ −0.50), which showed decreased enrichment in pathways related to epithelial development and cell differentiation (Figure 3D). Together, our results illustrate that in vitro LOX knockdown disrupts epithelial proliferation and differentiation.

### 3.4. Novel Murine Model of Epithelial Lox Knockout Demonstrates Altered Esophageal Proliferation and Differentiation

**Figure 4:**
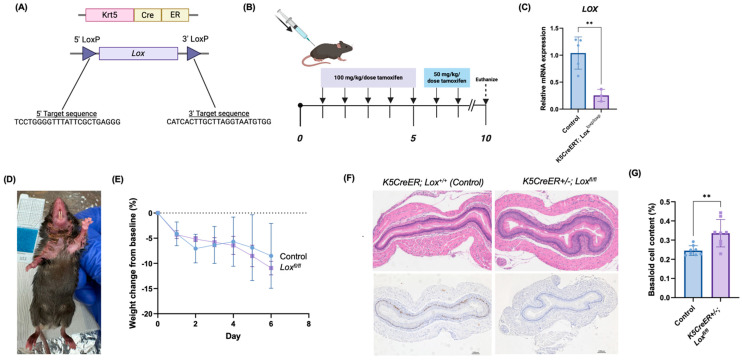
Murine model for depletion of epithelial Lox demonstrates decreased proliferation and basal cell hyperplasia. (**A**) Schematic of gene-targeting strategy to generate tamoxifen-induced *Lox*-knockout model specifically targeting stratified squamous epithelium. *LoxP* sites were inserted before exon 1 and after exon 3 in *Lox* gene. (**B**) Tamoxifen dosing schedule utilized to induce *K5CreER*. All mice received 100 mg/kg/dose tamoxifen for 5 days, followed by 50 mg/kg/dose for additional two days. Mice were euthanized on day 10 for analysis. (**C**) qRT-PCR demonstrating decreased *Lox* expression in *K5CreER; Lox^fl/fl^* mice compared to control mice. (**D**) Ventral hair loss pattern noted in *K5CreER;* *Lox^fl/fl^* mice on day 10. (**E**) Percent weight change from baseline for control versus *K5CreER;* *Lox^fl/fl^* mice. (**F**) H&E (**top**) and Ki-67 (**bottom**) staining on esophageal specimens from control versus *K5CreER;* *Lox^fl/fl^* mice. (**G**) Percent esophageal basaloid cell content, determined as percentage of total height of basaloid cell layers per esophageal diameter spanning both basaloid cell layers and differentiated cell layers, including keratinized inner cell mass in H&E-stained esophageal samples, with 3 areas measured per sample (control mice, n = 5; *K5CreER;* *Lox^fl/fl^* mice, n = 3). ** *p* < 0.01.

In order to validate our in vitro findings, we utilized a squamous epithelium-specific Lox-knockout murine model, *K5CreER+/−*; *Lox^fl/fl^,* with confirmed knockout after tamoxifen treatment (Figure 4A-C). Following tamoxifen exposure, the *K5CreER+/−*; *Lox^fl/fl^* mice demonstrated a spontaneous ventral hair loss pattern (Figure 4D). On day 10 post-tamoxifen, both the control and *K5CreER+/−*; *Lox^fl/fl^* mice exhibited consistent weight loss compared to their baseline weight, with no statistically significant difference in the percent weight loss between the two groups (Figure 4E).

The histological evaluation of esophageal sections from the *K5CreER; Lox^fl/fl^* mice revealed basal cell hyperplasia, illustrated by an increased basaloid cell content, compared to the control mice (Figure 4F,G). To assess basal cell proliferation in the esophageal epithelium of the control and *K5CreER+/−; Lox^fl/fl^* mice, we performed an IHC for Ki-67, a marker of cell proliferation. We found a significantly decreased abundance of Ki-67+ cells in the basal layer of the esophageal epithelium from the *K5CreER*; *Lox^fl/fl^* mice when compared to the basal layer from the control mice (Figure 4F). These findings suggest that an epithelial Lox knockout is associated with disrupted differentiation and proliferation in vivo.

## 4. Discussion

Epithelial differentiation is a tightly regulated process. Perturbed differentiation and impaired barrier function are implicated in several inflammatory conditions, including GERD, Barrett’s esophagus, and EoE. Improving our understanding of the molecular mechanisms underlying the homeostatic proliferation–differentiation gradient may facilitate better detection and therapy for these conditions. Here, we describe a novel critical role for LOX in esophageal epithelial development. We demonstrate that decreased LOX expression disrupts esophageal epithelial cell proliferation in two-dimensional cell culture and halts three-dimensional organoid formation. Furthermore, we show that LOX knockdown alters the expression of markers of epithelial differentiation, suggesting a disrupted epithelial proliferation–differentiation gradient. The transcriptomic analysis further supports the disruption of epithelial differentiation in the setting of in vitro LOX knockdown. Finally, we describe a novel murine epithelial Lox knockout model, which further demonstrates disrupted epithelial differentiation and proliferation in the esophagus. These results help to better define the role of LOX in the esophageal epithelium and suggest the integral role of LOX in epithelial renewal and maintenance of epithelial homeostasis.

Our findings build on prior work supporting a non-canonical role of LOX in epithelial differentiation. We have previously shown that in vitro LOX overexpression in esophageal epithelial cells enhances epithelial differentiation and barrier integrity [25]. Patient-derived esophageal epithelial organoids treated with IL-13 to induce barrier disruption demonstrated a marked upregulation of LOX expression in the differentiated epithelial cell population. Further, in vitro LOX overexpression in esophageal epithelial organoids promoted enhanced differentiation. Air–liquid interface cultures also demonstrated that LOX overexpression supports epithelial integrity and counteracts IL-13-induced barrier disruption. This prior work, in aggregate with the current report, proposes a non-canonical role for LOX as a signaling molecule important for esophageal epithelial differentiation, independent of its role in collagen crosslinking. Research on the skin has shown that in vitro LOX silencing in human keratinocytes similarly disrupts epidermal differentiation, resulting in increased expression of the early differentiation markers KRT10, involucrin, and transglutaminase 1 [24]. Notably, Le Provost et al. found that the irreversible pharmacologic inhibition of LOX enzymatic activity with 3-aminoproprionitrile fumarate (βAPN) did not result in altered keratinocyte differentiation, suggesting that LOX enzymatic activity was not necessary for the epidermal differentiation process [24]. Prior experimental studies evaluating the role of epithelial LOX in the esophagus and skin have relied heavily on cell culture techniques, given the lack of in vivo models for altered LOX expression. Global LOX deletion is embryonically lethal, resulting in structural alterations to the arterial walls and subsequent aortic aneurysms [16], previously making in vivo studies challenging. We have built on this prior body of work with our in vitro techniques, as well as with a novel murine model of epithelial LOX silencing.

In the cancer literature, LOX has been shown to drive metastasis and tumor growth [38]. In vitro models have shown that the production of LOX by mesenchymal stem cells leads to increased migration, metastasis, and resistance to anoikis of breast cancer cells [39,40]. Similarly, LOX can activate epidermal growth factor (EGF) signaling to drive tumor metastasis. The utilization of a LOX inhibitor decreased metastatic formation using in vivo breast cancer models [41]. In the liver, hepatic stellate cell production of LOX increases the metabolic fitness of cholangiocarcinoma in vivo, as demonstrated by inhibition studies [42]. Taken together, there is a growing body of evidence in the cancer literature that LOX directly impacts the growth and survival of tumors; however, its role in homeostasis is still underappreciated. In our in vitro studies, LOX is necessary for organoid formation, and LOX knockdown decreases cell metabolic activity and proliferative capacity. This suggests that LOX has an epithelial role in tissue renewal and growth during homeostasis.

Altered epithelial homeostasis is a histologic hallmark of a number of esophageal diseases, including EoE, where epithelial changes such as basal cell hyperplasia are a prominent feature [9,43]. Ongoing basal cell hyperplasia, even in EoE patients with histologically inactive disease based on eosinophil count, is associated with increased patient-reported symptoms and endoscopic findings [44]. We have previously shown that LOX is upregulated in the EoE esophagus, specifically in the esophageal epithelium [25,45], and that EoE-related cytokines, including TNFa, TGFb, and IL-13, all increase the expression of LOX in the epithelium [45]. The current therapies for EoE aim to decrease eosinophilic inflammation, but may not restore epithelial homeostasis at the molecular level [46]. Given its canonical role in collagen crosslinking, it is tempting to attribute the increased LOX expression in the EoE epithelium to its role in perpetuating fibrosis. Our current work suggests that finely regulated LOX expression may be integral to the maintenance of epithelial homeostasis, and increased epithelial LOX expression in active EoE, noted in our previous works [25,45], could reflect a response to epithelial injury.

To reconcile these two competing roles, we propose that epithelial injury may drive increased LOX expression in the esophageal epithelium to restore the epithelial proliferation–differentiation gradient. The subsequent migration of extracellular epithelial LOX to the lamina propria may inadvertently result in increased collagen crosslinking and the potentiation of fibrosis as a by-product. LOX has been proposed to play a similar “double-edged sword” role in muscular dystrophies, including Duchenne muscular dystrophy (DMD), where increased LOX expression is associated with a fibrotic phenotype, but also appears to modulate cell differentiation in both in vitro and in vivo models [47].

A limitation of our study is that a precise mechanistic pathway has not been delineated. A number of complex, tissue-specific interactions of LOX in relation to vascular, cardiac, pulmonary, dermal, and renal disorders have previously been identified, demonstrating that it can act as both a regulatory target and an active player in multiple signaling pathways [21]. We have previously proposed that LOX-mediated increases in bone morphogenetic protein 2 (BMP2) signaling drive downstream epithelial cell differentiation in EoE [25]. Future studies should aim to elucidate the underlying mechanism behind perturbations in epithelial proliferation and differentiation in the absence of LOX, as well as evaluate its impact on barrier integrity. Further, our current report utilized a single human cell line in vitro and an in vivo murine model to demonstrate uniform results. The use of a single in vitro cell line is a limitation of our study, and future work should validate these findings in additional cell lines.

## 5. Conclusions

Epithelial homeostasis is maintained by an exquisitely regulated proliferation–differentiation gradient. Herein, we describe a novel non-canonical role for LOX in the maintenance of proliferation and differentiation using both in vitro and in vivo models. Further investigations of epithelial LOX may provide novel insights into disease pathogenesis and potential therapeutic targets for a range of epithelial diseases, including EoE.

## Data Availability

Data are contained within the article and Supplementary Materials.

## Supplementary Materials

The following supporting information can be downloaded at: https://www.mdpi.com/article/10.3390/biom14121560/s1, Figure S1: Confirmation of doxycycline-induced LOX knockdown on mRNA and protein levels.
