# Supplementary material for: Lysyl Oxidase Mediates Proliferation and Differentiation in the Esophageal Epithelium
Source: Biomolecules. 2024 Dec 7;14(12):1560. doi: 10.3390/biom14121560 (PMC11674119; doi:10.3390/biom14121560)
Supplement: Supplementary file 1 [file biomolecules-14-01560-s001.zip › biomolecules-3313956-supplementary.pdf]

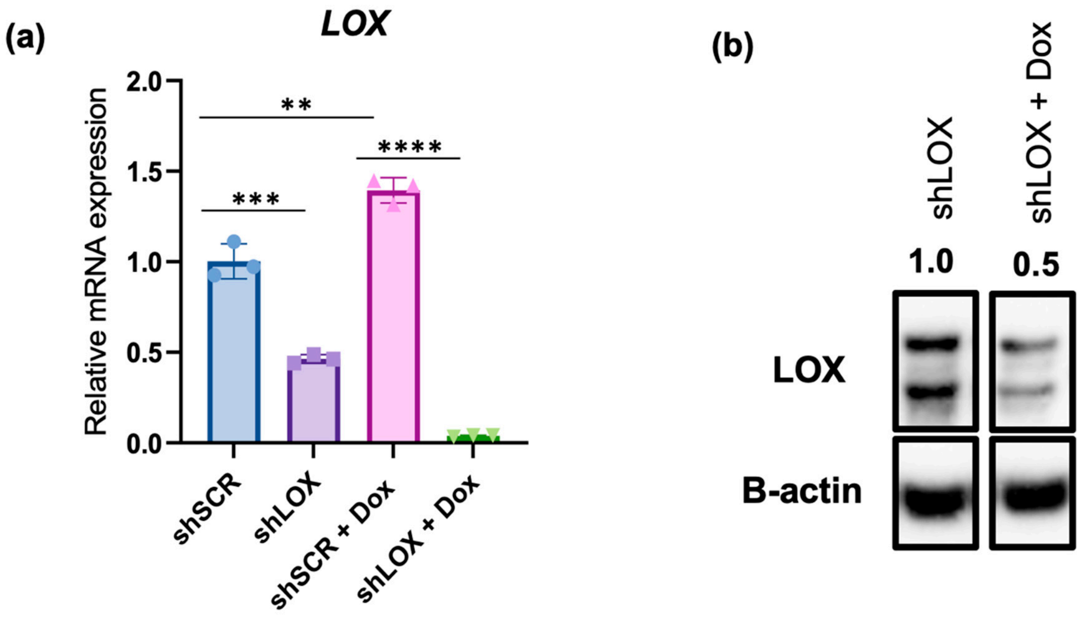

Supplementary Figure S1: Confirmation of doxycycline-induced LOX knockdown on mRNA and protein level. (a) qRT-PCR for LOX mRNA expression in monolayer-cultured EPC2-hTERT cells expressing doxycycline-inducible shRNA targeting scrambled mRNA (shSCR) versus LOX mRNA (shLOX) following with and without continuous doxycycline exposure. (b) Representative Western blot of Lox expression in monolayer-cultured EPC2-hTERT cells expressing doxycycline-inducible shRNA targeting scramble mRNA (shSCR) versus LOX mRNA (shLOX) following with and without continuous doxycycline exposure. Data are representative of 3 independent experiments and expressed as means ± SDs. ****$p<0.0001$; ***$p<0.001$; **$p<0.01$
